# Focus on Quality: Investigating Residents’ Learning Climate Perceptions

**DOI:** 10.1371/journal.pone.0147108

**Published:** 2016-01-14

**Authors:** Milou E. W. M. Silkens, Onyebuchi A. Arah, Albert J. J. A. Scherpbier, Maas Jan Heineman, Kiki M. J. M. H. Lombarts

**Affiliations:** 1 Professional Performance Research group, Center for Evidence-Based Education, Academic Medical Center/University of Amsterdam, Amsterdam, The Netherlands; 2 Department of Epidemiology, Fielding School of Public Health, University of California Los Angeles (UCLA), Los Angeles, California, United States of America; 3 UCLA Center for Health Policy Research, Los Angeles, California, United States of America; 4 Department of Educational Development and Research, Maastricht University, Maastricht, The Netherlands; University of California San Diego, UNITED STATES

## Abstract

**Background:**

A department’s learning climate is known to contribute to the quality of postgraduate medical education and, as such, to the quality of patient care provided by residents. However, it is unclear how the learning climate is perceived over time.

**Objectives:**

This study investigated whether the learning climate perceptions of residents changed over time.

**Methods:**

The context for this study was residency training in the Netherlands. Between January 2012 and December 2014, residents from 223 training programs in 39 hospitals filled out the web-based Dutch Residency Educational Climate Test (D-RECT) to evaluate their clinical department’s learning climate. Residents had to fill out 35 validated questions using a five point Likert-scale. We analyzed data using generalized linear mixed (growth) models.

**Results:**

Overall, 3982 D-RECT evaluations were available to investigate our aim. The overall mean D-RECT score was 3.9 (SD = 0.3). The growth model showed an increase in D-RECT scores over time (*b* = 0.03; 95% CI: 0.01–0.06; *p* < 0.05).

**Conclusions:**

The observed increase in D-RECT scores implied that residents perceived an improvement in the learning climate over time. Future research could focus on factors that facilitate or hinder learning climate improvement, and investigate the roles that hospital governing committees play in safeguarding and improving the learning climate.

## Introduction

Throughout the modernizations of postgraduate medical education (PGME), quality assurance (QA) and continuous quality improvement (QI) of residency training received considerable attention worldwide [1–3]. The department’s learning climate is considered to be an important indicator of PGME quality [2, 3] and as such often monitored as well as targeted during QI activities. However, whether these activities actually impact the learning climate is not clear.

The department’s learning climate (also known as the learning environment [4]) includes the formal and informal context in which learning takes place [5] and incorporates the perceived atmosphere of a department [6] as well as common perceptions of policies, practices and procedures [7]. It is acknowledged that a healthy learning climate contributes to the use of effective learning approaches [8], resident wellbeing [9–11] and training satisfaction [12]. Furthermore, the learning climate [10] is thought to influence residents’ perceptions of their own competencies [13], professional development [14] and resulting professional behavior. Hence, a healthy learning climate may benefit the development of the resident as a professional as well as the quality of care provided by the resident.

Recognition of the relevance of the learning climate has brought about an increase in QA/QI activities aimed at maintaining and improving learning climates in residency. As part of these activities, training programs often evaluate the learning climate by the administration of annual trainee surveys [1]. A widely used and well-researched questionnaire is the Dutch Residency Educational Climate Test (D-RECT) [15]. The D-RECT is a 35-item questionnaire evaluating nine domains of a department’s learning climate [16]. Repeated use of the D-RECT may provide insight into a department’s educational performance [17]. Ultimately, the results of a D-RECT evaluation might trigger QI initiatives aimed at enhancing the quality of the learning climate [15].

Given the importance of the learning climate for PGME quality and patient care, as well as the increase in QA/QI activities aiming to impact the learning climate, we investigated how the learning climate develops over time. The aim of our study is to investigate whether the D-RECT scores of training departments change over time.

## Methods

### Setting

In the Netherlands PGME is regulated by the Royal Dutch Medical Association. One of the core aspects of the regulations is the responsibility of clinical departments offering residency training to guarantee trainees a supportive learning climate [18]. The D-RECT is widely used throughout the Netherlands to continuously monitor the learning climate as perceived by residents. The aim of the D-RECT is to identify areas for improvement and, as such, serve as a foundation for QI initiatives.

### Design

For this study we used D-RECT learning climate data that were collected by teaching departments to evaluate the learning climate and to fuel QA/QI initiatives. Departments that wished to evaluate the learning climate could request and perform a D-RECT evaluation via a web-based system. In order to investigate the change of D-RECT scores over time, we assessed the longitudinal development of the scores.

### Participants and data collection

All residents in clinical teaching departments that requested a D-RECT evaluation were invited to complete the questionnaire during a pre-determined period (commonly one month). Residents received a maximum number of three automatically generated reminders by e-mail. The number of residents differed per residency training program. We included departments that used the D-RECT at least once between January 2012 and December 2014. Departments originated from academic hospitals (providing top clinical care, scientific research and PGME as well as coordinating PGME for affiliated hospitals), top clinical teaching hospitals (providing top clinical care, scientific research and PGME) or general teaching hospitals (providing patient care and PGME). Participation in the D-RECT was voluntary and anonymous for all residents.

We obtained informed consent for the use of the D-RECT data. The institutional ethical review board of the Academic Medical Center of the University of Amsterdam provided a waiver stating the Medical Research Involving Human Subjects Act (WMO) did not apply to the current study.

### The D-RECT

The D-RECT was originally developed and preliminarily validated by Boor et al [15]. Recently, the original D-RECT was updated and extensively validated, leading to a 35-item questionnaire, covering nine domains: *educational atmosphere*, *teamwork*, *role of specialty tutor*, *coaching and assessment*, *formal education*, *resident peer collaboration*, *work is adapted to residents’ competence*, *accessibility of supervisors*, *and patient sign-out* [16]. The items of the D-RECT can be answered on a five point Likert-scale (1 = totally disagree, 2 = disagree, 3 = neutral, 4 = agree, 5 = totally agree). Additionally, a “not applicable” option is provided.

### Data analysis

Descriptive statistics and frequencies were used to describe the main characteristics of the study population. Departments with less than three resident evaluations were removed from the analysis since previous research showed that at least three evaluations are needed for a reliable mean total score of the D-RECT [16]. For departments that used the D-RECT more than once a year, only the most recent measurement period was included. Resident evaluations that were missing more than 17 questions (>50%) were excluded from further analysis. For the remaining evaluations, missing values were assumed to be missing at random and imputed by using expectation-maximization (EM). An average composite score representing the overall learning climate was computed for the 35 items of the D-RECT.

To investigate whether the D-RECT scores of clinical departments change over time, individual resident evaluations were aggregated at the department level to get each department’s annual mean score. Unadjusted and adjusted linear growth models were used to assess the growth of D-RECT scores over time [19]. By using a linear mixed model with random intercept, the analysis accounted for the hierarchical clustering of repeated scores within departments and of departments within teaching hospitals. For the adjusted models, the type of hospital (academic, top clinical teaching hospitals, general hospital), the number of resident evaluations aggregated to a department score, gender of respondents and the year of training were used as covariates. Besides residents, we decided to include D-RECT evaluations provided by doctors not in training and fellows as well. Since program directors selected respondents they considered relevant to evaluate the department's learning climate, we decided to follow the program directors' decisions and include these trainees and fellows. To check the results, we performed the analyses with additional summary outcome definitions, namely median composite scores and factor scores [20].

Resulting associations were reported using regression coefficients (*b*) and their 95% confidence interval (95% CI). All analyses were performed using SPSS Statistics version 20 (IBM Corp).

## Results

### Study participants

In total, 4190 residents completed the D-RECT questionnaire between January 2012 and December 2014. After exclusion due to missing values, number of evaluations or double measurements in one year, a sample of 3982 evaluations remained. The overall response rate was 70%, varying from 24% to 100% between departments. The sample represented 223 training programs in 39 teaching hospitals. The number of training programs that participated per hospital varied between 1 and 27. Of the 3982 evaluations, 1244 (31.2%), 1376 (34.6%) and 1362 (34.2%) depict the learning climate in 2012, 2013 and 2014 respectively. A detailed description of the study sample is provided in Table 1.

**Table 1:** Characteristics of the study population.

Characteristics	Study sample
Number of resident evaluations, n	3982
Male residents, n (%)	1615 (40.6)
Female residents, n (%)	2363 (59.3)
Missing, n (%)	4 (0.1)
Number of training programs evaluated, n	223
Number of training programs per hospital, n	
1	17
2	4
3	1
4	3
5	1
6	4
10	2
13	2
14	1
18	1
23	1
26	1
27	1
Number of teaching institutes, n	39
Academic, n (%)	4 (10.3)
General, n (%)	21 (53.8)
Top clinical teaching hospitals, n (%)	14 (35.9)
Year of training, n (%)	
1	678 (17)
2	738 (18.5)
3	606 (15.2)
4	524 (13.2)
5	409 (10.3)
6	238 (6)
Doctor not in training	717 (18)
Fellow	72 (1.8)
Missing	4 (0.1)
Overall learning climate score, mean (SD)	3.9 (0.3)

SD: standard deviation

### Learning climate change over time

The overall D-RECT score in the sample was 3.9 (Table 1). Mean D-RECT scores showed modest increases from 3.83 in 2012 to 3.86 in 2013 and to 3.91 in 2014 in the overall sample (Table 2). The unadjusted and adjusted growth models indicated this change was statistically significant (*b* = 0.03; 95% CI = 0.01–0.06; *p* < 0.05) (Table 3).

**Table 2:** Mean learning (D-RECT) scores in 2012, 2013 and 2014.

	Mean score 2012	Mean score 2013	Mean score 2014
Mean learning climate score in the full sample	3.83 (0.33)	3.86 (0.30)	3.91 (0.28)

**Table 3:** Unadjusted and adjusted growth models for annualized change in learning climate (D-RECT) scores.

	Unadjusted model		Adjusted model	
	Regression coefficient (95% CI)	P-value	Regression coefficient (95% CI)	P-value
Change-in-score in the full sample	0.03 (0.01–0.06)	<0.01	0.03 (0.01–0.06)	<0.01

## Discussion

### Main findings

Our study showed that from 2012 to 2014 the learning climate in residency training, as measured using the D-RECT questionnaire, improved significantly (from 3.83 to 3.91 on a 5-point scale) in an overall sample totaling 223 training programs.

### Explaining learning climate change over time

Regarding our aim (to investigate whether the learning climate perceptions of residents change over time) our findings suggest a positive trend in the D-RECT scores. This implies that residents experienced an improvement in the learning climate. However, the absolute improvement in learning climate is quite small. Both instrument-based and practice-based arguments can be provided to explain these results. Regarding the instrument, small effects might be caused by properties of the answer scale [21]. The D-RECT makes use of a 5-point Likert scale, which gives residents a limited range of responses. As a result, respondents are restricted in their ability to indicate improvement and have few options to discriminate between levels of performance [21]. Research into the evaluation of surgeons’ teaching performance with a 5-point Likert scale instrument demonstrated a comparable small but positive effect [22].

From a practice-based perspective the progress measured may be a reflection of the increased attention paid to the quality of residency training at various levels in the Dutch health system. The introduction of competency based medical education has elicited numerous changes in PGME, including new regulations that have ratified the importance of healthy learning climates for residents' learning and for patient care. QA/QI efforts undertaken by hospital and departmental leadership over the past few years are likely contributors to the progress evidenced in this study. In particular it is known that climate is relatively resistant to change. Although the use of the D-RECT in the Netherlands was initiated in 2009, wide-scale spread of the instrument only started in 2012. As demonstrated in previous research, it may take a couple of years after the start of evaluation before a convincing improvement in the learning climate becomes noticeable [23]. Therefore, the identified small but positive trend towards improvement of the learning climate within the studied time span can be considered encouraging.

A second practice-based explanation for the small change might be that departments are not (yet) acting from an improvement perspective. Ideally, departments will work towards improvement of the learning climate on a continuous basis. Whereas departments with lower scores might feel pressured to use their data to improve the learning climate, clinical departments with high D-RECT scores might have less incentive to initiate QI actions. The learning climate evaluations in our sample were quite high (mean D-RECT scores above 3.83) and suggested that most residents perceived the learning climate as healthy. Faculty and residents might have interpreted these high scores to mean that no further action towards improvement was needed, resulting in limited progress of the learning climate.

### Strengths and limitations of the study

We were able to use the widely accepted [24, 25] and well researched [16] D-RECT to assess the learning climate in PGME. Due to the wide spread use of the instrument in the Netherlands, we could use a large pool of resident evaluations to assess change over time. Although there is no consistent sampling rule for multilevel models, a general recommendation is to have 20 higher- and 20 lower-level units of analysis [26]. Although our sample had over 20 higher-level units, we made sure to use restricted maximum likelihood during estimation to reduce the impact of the number of higher-levels [26].

Furthermore, a common statistical phenomenon when analyzing repeated measures is regression to the mean [27], which tends to make the observation of change in data more likely. The design of the current study limited the opportunities for analyzing whether regression to the mean is present in the data and therefore its possible contribution to the observed changes in D-RECT scores cannot be completely dismissed.

With regards to the generalizability of this study, the multicenter approach of the current study, with the inclusion of both academic teaching hospitals as well as top clinical and general teaching hospitals all over the Netherlands, contributed to the representativeness of the study population.

### Implications for practice and future research

Our results suggest that the recent focus on the learning climate may have resulted in a small statistically significant improvement in the learning climate for PGME in the Netherlands. To continuously improve the learning climate, departments could use the D-RECT results for defining QA/QI initiatives and setting improvement goals. In the Netherlands, some departments have been successful by giving residents a leading role in these efforts.

In general, the frequent use of (various) feedback generating tools can support a department's monitoring activities regarding the quality of residency training and learning climate in particular [23]. In the Netherlands, legislation enacted in 2011 underlines and ratifies this approach and hospital-wide committees are held accountable for its execution. Hospital-wide monitoring committees (HMCs), which are mandatory for each teaching hospital, represent all residency program directors, residents, and the hospital board [18], but may also represent other staff. The aim of the HMC is to oversee PGME quality and support the QA/QI initiatives of all training programs in a teaching hospital [18]. The HMC may serve as a platform for sharing best practices regarding QI/QA initiatives and thus facilitate the exchange of ideas between departments [28]. A well functioning HMC is expected to contribute to improved departmental learning climates and, ultimately, improved training outcomes. Future research will have to prove these intended effects.

Although the relevance of bodies such as the HMC has been stressed, previous research showed that the systematic approaches used by the HMCs in 2011 were premature [29]. Since evidence shows systematic organizational policies contribute to PGME quality [28, 30], more qualitative research should be undertaken to explore the factors that hinder or support the use of a systematic approach. Furthermore, we are aware that besides QA/QI initiatives, there might be numerous other factors influencing the department’s learning climate (e.g. organizational culture). Therefore, investigating the mechanism underlying learning climate change remains important.

## Conclusions

This study provides insight into the development of the learning climate over time, suggesting that residents perceive an improvement in the learning climate. Future research could focus on factors that facilitate or hinder learning climate improvement, including the role of hospital governing committees.

## Supporting Information

S1 Dataset (SAV)
